# 873. Comparison of Antibiotic and Antiviral Use before and after Deployment of Rapid Influenza Testing in the Emergency Department

**DOI:** 10.1093/ofid/ofac492.066

**Published:** 2022-12-15

**Authors:** Alec Wesolowski, Jennifer D Clifton, Jessica Miller, Maureen Shields

**Affiliations:** Advocate Lutheran General Hospital, Chicago, Illinois; Advocate Lutheran General Hospital, Chicago, Illinois; Advocate Lutheran General Hospital, Chicago, Illinois; Advocate Aurora Health, Chicago, Illinois

## Abstract

**Background:**

Influenza shares common symptoms with bacterial pneumonia, resulting in inappropriate prescribing of antibiotics that may lead to increased risk of adverse reactions, antimicrobial resistance, and healthcare costs. Rapid influenza polymerase chain reaction (PCR) tests have reduced turnaround times compared to multiplex PCR respiratory panels allowing for earlier diagnosis, which may improve antimicrobial stewardship outcomes in the emergency department (ED). This study aims to compare antibiotic and antiviral use before and after deployment of a rapid influenza PCR in the ED.

**Methods:**

This single-center, retrospective, cohort study included pediatric and adult patients discharged from the ED with a positive influenza test with either the multiplex PCR respiratory panel (January 2017 – July 2019) or rapid PCR (July 2019 – February 2020). The primary endpoint was antibiotic use pre- and post-implementation of the rapid influenza PCR in the ED. Secondary endpoints included antiviral use, duration of antimicrobial therapy, test turnaround time, ED length of stay, 30-day readmission, and adverse events. A multivariable regression model evaluated patient factors associated with antimicrobial prescribing.

**Results:**

A total of 620 positive influenza results were identified with 280 patients (multiplex PCR = 33; rapid PCR = 247) meeting inclusion criteria. Patients were less likely to be prescribed antibiotics (39.4% vs 8.9%, OR 0.15, 95% CI 0.067–0.34) and more likely to be prescribed antivirals (24.2% vs 61.1%, OR 4.92, 95% CI 2.13–11.34) with the rapid influenza PCR. Rapid influenza PCR significantly reduced ED length of stay (4.9 vs 3.4 hours, p < 0.01) and test turnaround time (27 hours vs 3.5 hours, p < 0.01). Patients at high risk for complications associated with influenza were more likely to be prescribed antiviral therapy (25% vs 77.7%, OR 5.27, 95% CI 3.02–9.17). Based on the regression analysis conducted, asthma, (OR 3.5, 95% CI 1.48–8.26), immunosuppression (OR 9.6, 95% CI 1.18–78.2), and age less than 5 years old (OR 3.1, 95% CI 1.80–5.45) are predictors of antiviral prescribing.

**Conclusion:**

Implementation of a rapid influenza PCR in the ED reduced antibiotic use and optimized antiviral therapy for patients at higher risk of influenza with complications.

**Disclosures:**

**All Authors**: No reported disclosures.

